# Hippocampal volume reduction in children with chromosome 22q11.2 deletion syndrome is associated with cognitive impairment

**DOI:** 10.1186/1744-9081-3-54

**Published:** 2007-10-23

**Authors:** Tracy DeBoer, Zhongle Wu, Aaron Lee, Tony J Simon

**Affiliations:** 1 M.I.N.D. Institute, Psychiatry and Behavioral Sciences, University of California, Davis, USA

## Abstract

**Background:**

Previous investigations of individuals with chromosome 22q11.2 deletion syndrome (DS22q11.2) have reported alterations in both brain anatomy and cognitive function. Neuroanatomical studies have reported multiple abnormalities including changes in both gray and white matter in the temporal lobe, including the amygdala and hippocampus. Separate investigations of cognitive abilities have established the prevalence of general intellectual impairment, although the actual extent to which a single individual is affected varies greatly within the population. The present study was designed to examine structures within the temporal lobe and assess their functional significance in terms of cognition in children with DS22q11.2.

**Method:**

A total of 72 children (ages 7–14 years) participated in the investigation: 36 children (19 female, 17 male) tested FISH positive for chromosome 22q11.2 deletion (Mean age = 10 years 9 months, ± 2 yr 4 mo) and 36 were age-matched typically developing controls (13 female, 23 male; Mean age = 10 years 6 months, ± 1 yr 11 mo). For each subject, a three-dimensional high-resolution (1 mm isotropic) T1-weighted structural MRI was acquired. Neuroanatomical guidelines were used to define borders of the amygdala and hippocampus bilaterally and volumes were calculated based on manual tracings of the regions. The Wechsler Intelligence Scale for Children (WISC) was also administered.

**Results:**

Volumetric reductions in total gray matter, white matter, and both the amygdala and hippocampus bilaterally were observed in children with DS22q11.2. Reductions in the left hippocampus were disproportionate to decreases in gray matter after statistically controlling for group differences in total gray matter, age, and data collection site. This specific reduction in hippocampal volume was significantly correlated with performance on standardized measures of intelligence, whereas the other neuroanatomical measures were not (gray/white matter, CSF, and amygdala).

**Conclusion:**

Results from this study not only contribute to the understanding of the neuroanatomical variation in DS22q11.2, but also provide insight into the nature and source of the cognitive impairments associated with the syndrome. Specifically, we report that decreases in hippocampal volume may serve as an index of severity for cognitive impairments in children with DS22q11.2.

## Background

Chromosome 22q11.2 deletion syndrome (DS22q11.2) is a congenital condition known to affect brain development and cognition that occurs in 1–2 out of every 4,000 live births [1]. The syndrome results from a microdeletion on the long arm (q) of the 22^nd ^chromosome and has been identified as the molecular cause for several symptom-based medical diagnoses [2]. Medical characteristics associated with the syndrome typically include palatal abnormalities and/or velopharyngeal insufficiency, immune deficiency, congenital cardiac defects, neonatal hypocalcemia, and facial dysmorphisms [3,4]. Individuals with DS22q11.2 are also at an increased risk for developing psychiatric disorders such as ADHD [5], obsessive-compulsive disorder [6], affective disorders [7], autism [5], and schizophrenia [8]. Significant intellectual impairments are often observed as well [9]. However, at present, far less is known about the cognitive characteristics than for the medical indications.

Impairment in general intellectual functioning is one of the most consistently reported features in DS22q11.2 [9]. IQ scores typically fall in the range of 70–85, one full standard deviation or more below the population mean. Empirical investigations have demonstrated that low performance on IQ assessments are not the result of the physical or medical characteristics [10,11], nor the associated behavioral problems that commonly accompany the syndrome [12]. Studies of academic achievement have attempted to further clarify these cognitive difficulties and report that children with DS22q11.2 perform better at reading and spelling than arithmetic [13]. This specific pattern of cognitive strengths and weaknesses has lead to the hypothesis that the cognitive difficulties are the cascaded effects of core deficits in visuospatial processing and attention, both of which are primarily mediated by cortical networks in the parietal and temporal lobes [14-16].

At least some of the genetic material in the deleted segment (30–40 genes) appears to be related to typical brain development, as anomalous brain structure is consistently reported [17,18]. Thus far, characterization of the expression of DS22q11.2 in terms of neurodevelopment has come from studies using structural and function magnetic resonance imaging and diffusor tensor imaging in children, adolescents, and adults with the syndrome [18-21]. The most reliable neuroanatomical finding thus far is an overall reduction in total brain volume ranging from 8.5–11% that appears more concentrated in the posterior and inferior regions of the brain [19-21]. During childhood, volumetric reductions in the temporal lobe have been reported as the result of decreases in both gray and white matter [22]. Similarly, studies in adulthood also report reductions in temporal lobe volume, with greatest reductions emerging in the presence of marked psychopathology [18,23].

Although the number of studies examining individual structures (i.e., amygdala and hippocampus) in the temporal lobes is increasing, findings to date have been variable (cf. [22,24-26]). Some of this heterogeneity can be attributed to small sample sizes, differences in the ages, cognitive abilities, and psychiatric symptoms of those tested, the control groups chosen for comparison, the methods used to determine anatomical boundaries, and the covariates used in analyses. For example, disproportionate decreases in hippocampal volume have not typically been reported when total brain volume is used as a covariate (e.g., [22,26]), but have been reported when total gray matter is used (e.g., [24,25]). Moreover, these findings are difficult to compare directly given the wide range of ages and cognitive abilities of the participants, as well as variations in the tracing methods used for volumetric analyses (cf.; [27,28]).

Thus, although some progress has been made in characterizing the neuroanatomical differences within this population, the implications for neurocognitive outcomes remain poorly understood. Bearden and colleagues [17] have begun to address this issue by relating regional brain abnormalities with cognitive ability and behavioral phenotype in a sample of 13 children with DS22q11.2. Results of this investigation indicated that only temporal lobe volume (both gray and white matter) significantly predicted overall cognitive performance in children with DS22q11.2 [17]. Specifically, temporal lobe volume was a significant positive predictor of Full Scale and Verbal IQ, but not Performance IQ (as measured by WISC-III). In addition, Bearden et al. [17] report a negative correlation between Thought Problems as assessed by the Child Behavior Checklist (CBCL; [29]) and temporal lobe gray matter volume.

Within the temporal lobe are two structures on which much empirical research has been conducted: the amygdala (implicated in emotion and social behavior) and the hippocampus (well-known for its role in memory and spatial cognition). Investigation of these individual structures may provide more detail regarding regionally specific changes within the temporal lobe network. While the hippocampus has been measured volumetrically in DS22q11.2 and has been hypothesized to be related to risk for psychopathology [22] and memory impairments [25], to our knowledge, no evidence exists to date regarding the functional significance resulting from these observed abnormalities. Studies with typically developing children have suggested that the hippocampus is correlated with both IQ [30] and memory (see [31] for review). Also relevant for studies of children with DS22q11.2, there is evidence of associations between reductions in hippocampus volume in schizophrenia (see [32] for review), which may make assessment of this structure important for risk assessment and prediction of outcomes.

In short, the purpose of present investigation was to expand on previous research examining neuroanatomical differences in children with DS22q11.2 and link these findings to resulting cognitive outcomes. The ultimate goal of this line of research is to gain further insight into complex gene-brain-behavior relations associated with this syndrome and improve outcome predictions. Volumetric measurements of regions within the temporal lobe, namely the hippocampus and amygdala, were measured bilaterally from structural MRIs in 72 children and adolescents with and without DS22q11.2. Associations with intellectual ability were assessed.

## Methods

### Participants

A total of 72 children (ages 7–14 years) participated in this investigation: 36 children (19 female, 17 male) diagnosed with chromosome 22q11.2 deletion syndrome (Mean age = 10 years 9 months, ± 2 yr 4 mo) and 36 age-matched typically developing controls (13 female, 23 male; Mean age = 10 years 6 months, ± 1 yr 11 mo). Informed asset and consent was obtained from all participants and their guardians prior to participation. Diagnosis of chromosome 22q11.2 deletion was confirmed using fluorescent in situ hybridization (FISH). The experimental protocol followed guidelines set forth in the Helsinki Declaration for the ethical treatment of human research participants and was approved by the governing Institutional Review Boards of the data collection sites (Children's Hospital of Philadelphia, Hospital of the University of Pennsylvania, and the University of California, Davis Medical Center).

### Procedure

MRI data were acquired using a 1.5T Siemens Vision and two 3T Siemens Trios (Siemens Medical Solutions, Welangen, Germany). For each subject, a three-dimensional high-resolution (1 mm isotropic voxels) structural MRI was acquired using a T1-weighted MP-RAGE sequence. Before MRI scanning, children received head motion suppression training in the laboratory and in a mock MRI scanner. Scanner parameters were as follows: 1.5T Siemens Magnetom Vision scanner (Siemens Medical Solutions, Erlangen, Germany), TR = 9.7 ms, TE = 4 ms, 12 degree flip angle, number of excitations = 1, matrix size, 256 × 256, slice thickness = 1.0 mm, yielding 160 sagittal slices with an in-plane resolution of 1 × 1 mm, 3T Siemens Magnetom Trio, TR = 1820 ms, TE = 2.93 ms, 12 degree flip angle, number of excitations = 1, matrix size, 256 × 256, slice thickness = 1.0 mm, yielding 160 axial slices with an in-plane resolution of 1 × 1 mm, 3T Siemens Magnetom Trio, TR = 1620 ms, TE = 3.87 ms, 15 degree flip angle, matrix size = 256 × 192, slice thickness = 1.0 mm, 160 axial slices with in-plane resolution of 0.98 × 0.98 mm. Although data collection site was not significantly correlated with any of the volumetric measurements it was entered as a covariate in all analyses.

In addition to the structural MRI procedure, the Wechsler Intelligence Scale for Children (WISC-III/IV) was also administered to 41 participants (20 control, 21 Ds22q11.2). In order to equate WISC versions IIII and IV Verbal Comprehension Factor/Perceptual Organization (nonverbal) Factor (VC/PO) were used from WISC-III and Verbal Comprehension Index/Perceptual Reasoning Index (VCI/PRI) were used from WISC-IV. The remaining children were either unable to return for clinical evaluation or received an abbreviated version of the intelligence assessment which was not compatible with the WISCII-IV. Neuroanatomical findings for this subgroup of 41 children comparing amygdala and hippocampal volume were identical to that of the entire sample.

### Data analysis/processing

Overall brain volume measurements for each tissue type (gray matter, white matter, and cerebrospinal fluid or CSF) were calculated using the segmentation algorithms in SPM2 (Wellcome Department of Cognitive Neurology, London, UK, [33]). All MRI scans were spatially normalized through registration to a template before the segmentation was preformed. For the spatial normalization, a pediatric brain template (CCHMC2 template, Cincinnati Children's Hospital Medical Center, Cincinnati, OH) was used instead of the default ICBM's adult template in SPM2 as we wanted to minimize the amount of deformation during the non-linear spatial normalization for our child participants [34]. The CCHMC2 template was generated from 200 healthy children, 98 boys and 102 girls, ranging in age from 5 to 18 years (mean age was 11.43 F 3.59 years) at the date of the MR-exam. The registration between individual subjects and the template was performed by minimizing the residual sum of squared difference with a 12-parameter affine transformation followed by a non-linear transformation comprising a linear combination of 7 × 8 × 7 smooth spatial basis function. The spatially normalized images were then resliced to a resolution with voxel size of 2 × 2 × 2 mm.

Tissue probability values for gray matter, white matter, and CSF were generated using SPM's segmentation algorithm. Voxelwise Bayesian classification based on individual image intensity and prior probability maps (i.e., CCHMC2 maps) was iteratively computed followed by bias correction. After segmentation, some non-brain voxels, such as scalp, skull and venous sinuses, and odd voxels on tissue edges were cleaned from the segmented images by using a series of fully automated morphological operations [35]. The segmented tissue maps were modulated by the Jacobian determinants derived from the spatial deformation field to preserve the total brain volume. Total volumes for gray matter, white matter and CSF were then obtained by summing the voxel values in the modulated images.

**Figure 1:**
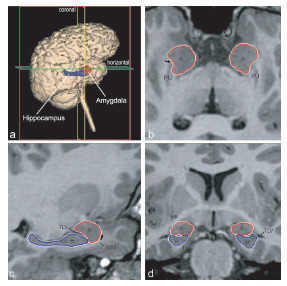
Orthogonal views for segmenting the amygdala and hippocampus on MRI sections. A three dimensional reconstruction of images (a) in which lines indicate the position of the horizontal plane (b), sagittal plane (c), and coronal plane (d) is shown. The arrow in b indicates the best-fit line along the white matter separating the amygdala from the putamen; the arrow in c represents the white matter that forms the ventral border of the rostral amygdala. A, Amygdala; EC, entorhinal cortex; H, hippocampus; PU, putamen; TLV, temporal horn of the lateral ventricle; WM, subamygdaloid white matter. Figure reproduced with permission from [37]. Copyright 2004 by the Society for Neuroscience.

For manual tracings of the amygdala and hippocampus, non-normalized T1-weighted images were imported into Analyze 5.0 (Biomedical Imaging Resource, Rochester, MN; [36]) and converted to cubic voxel dimensions of 0.469 mm (using a cubic spline interpolation algorithm). Images were reoriented so that the horizontal axis was parallel to a line from the rostral to the caudal pole of the hippocampus. Coronal sections were viewed perpendicular to the horizontal axis. On each coronal image, neuroanatomical guidelines were used to define borders of the amygdala and hippocampus bilaterally and volumes were calculated based on manual tracings of the regions in native space, see Figure 1 (complete details of the procedure can be found in [37]).

After establishing inter-rater reliability of greater than .90 on 10 training cases, raters who were blind to group status manually traced the amygdala and hippocampus. Approximately 10% of the cases included in this investigation were then re-traced by all raters to ensure equality between tracings. Inter-rater reliability for the left and right amygdala was: .952 and .949; inter-rater reliability for the left and right hippocampus was: .953 and .923.

## Results

Multivariate ANCOVAs were used to assess group differences in gray and white matter and CSF, with age and data collection site entered as covariates. Multivariate ANCOVAs were also used to assess group differences in both right and left amygdala and hippocampus volumes, with age, data collection site, and gray matter volume entered as covariates. Analyses regarding bilateral volume of the amygdala and hippocampus were rerun using total brain volume as a covariate in place of gray matter volume. Results did not differ with regards to the specific covariate used, thus only the former are reported. Data points from two outliers were removed from the analyses of the hippocampus and amygdala to ensure volumetric data met the necessary criteria for employing parametric statistics (i.e., assumption of normality and homogeneity of variance between groups).

Results indicated that, after statistically controlling for possible differences due to data collection site and age, the DS22q11.2 group had significantly less gray (M = 710.58 cm^3^, SD = 67.97) and white (M = 356.51 cm^3^, SD = 51.10) matter compared to controls (M = 763.63 cm^3^, SD = 72.48 and M = 388.46 cm^3^, SD = 46.32), *Fs*(1, 68) = 10.12, 8.6 respectively, *p*s < .01. However, CSF volume did not differ between the groups (DS22q11.2 M = 188.36 cm^3^, SD = 50.16, control M = 198.56 cm^3^, SD = 37.22, *F*(1, 68) = .66, *p *= .49). After statistically controlling for differences in gray matter, age, and data collection site, there were no significant differences in bilateral volumes of the amygdala between DS22q11.2 (left hemisphere: M = 1.76 cm^3^, SD = 2.78, right hemisphere: M = 1.77 cm^3^, SD = 2.56) and control (left hemisphere M = 1.90 cm^3^, SD = 2.50, right hemisphere: M = 1.90 cm^3^, SD = 2.50) groups, *F*s(1, 65) = 2.08, 0.66, *p*s = .15, .24 respectively. After controlling for differences in gray matter, age, and data collection site, left hippocampal volume was significantly reduced in children with DS22q11.2 (M = 2.31 cm^3^, SD = 3.11) compared to controls (M = 2.56 cm^3^, SD = 3.73), *F*(1, 65) = 7.29, *p *<.01. However, right hippocampal volume did not differ between the DS22q11.2 group (M = 2.46, SD = 3.24) and controls (M = 2.57 cm^3^, SD = 3.45), *F*(1,65) = .28, *p *= .60. Results for the cognitive assessment indicated that, as a group, DS22q11.2 scored significantly below controls on all IQ indices, see Table 1.

**Table 1:** Mean IQ (SD) for DS22q11.2 and control groups

	Controls (n = 20)	DS22q11.2 (n = 21)	One-way ANOVA
VC/VCI	111 (11)	81 (14)	*F*(1, 39) = 61.01, *p *< .001
PO/PRI	112 (11)	78 (13)	*F*(1, 39) = 82.99, *p *< .001
FSIQ	110 (11)	77 (12)	*F*(1, 39) = 88.70, *p *< .001

**Figure 2:**
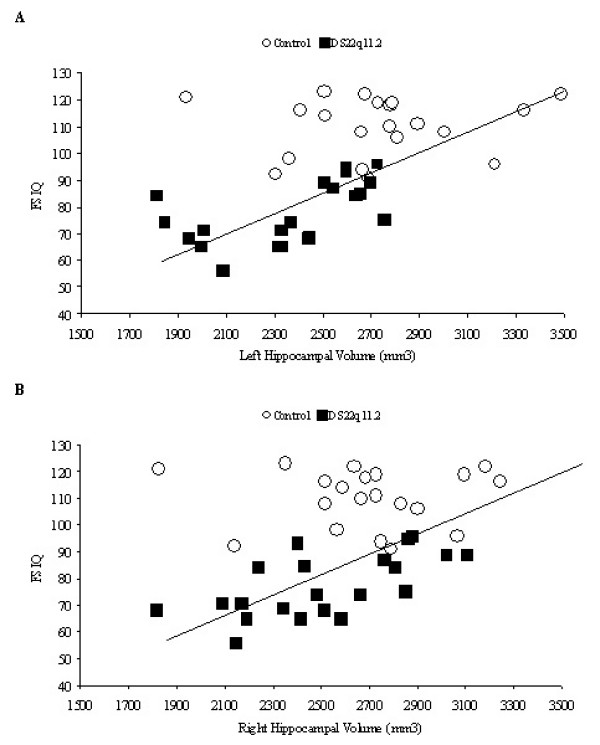
Linear regression scatterplot showing that left (a) and right (b) hippocampal volume measured in cubic millimeters (mm^3^) positively correlates with Full Scale IQ (r^2 ^= .32 and .15 respectively)

In order to determine if differences in neuroanatomical measurements were related to differences in cognition, correlational analyses were conducted between IQ (as measured by WISCIII/IV) and tissue volumes. The pattern of results was identical when z-score transformations were used in place of tissue volumes. Thus, for the sake of brevity, only the results from the non-transformed dataset are presented. Hippocampal volume was significantly correlated with performance on standardized measures of intelligence; however, other cortical measures (gray/white matter, CSF, and left and right amygdala) were not. Table 2 presents the results of the bivariate correlations and an illustration of association between hippocampus and FSIQ is presented in Figure 2.

**Table 2:** Two-tailed bivariate correlations of neuroanatomical and IQ measures across both DS22q11.2 and control groups

	Gray Matter	White Matter	CSF	Amygdala		Hippocampus	
				Left	Right	Left	Right
VC/VCI							
Pearson's r	0.19	0.19	-0.12	0.24	0.17	.62***	.50***
PO/PRI							
Pearson's r	0.17	0.18	0.02	0.25	0.21	.58***	.47***
FSIQ							
Pearson's r	0.11	0.08	-0.19	0.16	0.13	.56***	.39**

**p ≤ .01

*** p ≤ .001

All correlations between IQ and hippocampal volume remained statistically significant after entering age, gray matter, and data collection site as covariates (left hemisphere VC/VCI r(36) = .61, *p *< .001, PO/PRI r(36) = .61, *p *< .001, FSIQ r(36) = .61, *p *< .001, right hemisphere VC/VCI r(36) = .45, *p *< .01, PO/PRI r(36) = .45, *p *< .01, FSIQ r(36) = .42, *p *< .01). Partial correlations for each group are presented in Table 3. In order to determine if a specific subtest of the IQ assessments contributed more than the others to the reported effects, partial correlations were computed on four common subtests (Similarities, Vocabulary, Block Design, and Comprehension). Results of the bivariate correlational analyses are presented in Table 4. Subtest scores for 6 individuals could not be obtained from the clinic with which we were collaborating and thus were not included in the follow-up analysis. Across groups, performance on all four subtests was correlated with hippocampal volume. However, these correlations were not significant when groups were analyzed separately. This finding suggests that performance on one individual subtest was not solely responsible for the overall result of associations between hippocampal volume and IQ.

**Table 3:** Two-tailed partial correlations of hippocampus and IQ measures for both DS22q11.2 and control groups

	Controls (n = 20)		DS22q11.2 (n = 21)	
	Left	Right	Left	Right
VC/VCI				
Pearson's r	0.30	0.37	.49*	.62**
PO/PRI				
Pearson's r	.60**	.57*	0.25	0.42†
FSIQ				
Pearson's r	0.38	0.31	.48*	.58*

† p < .10

* p < .05

** p ≤ .01

**Table 4:** Two-tailed partial correlations of hippocampus and IQ subtests for DS22q11.2 and control groups combined

	Left	Right
Similarities		
Pearson's r	.47***	0.29†
Vocabulary		
Pearson's r	.54***	.40*
Block Design		
Pearson's r	.53**	.39*
Comprehension		
Pearson's r	.51**	.50**

† p < .10

* p < .05

** p ≤ .01

*** p < .001

In sum, within the DS22q11.2 group we report significant reductions in hippocampal volume that, in the left hemisphere, were disproportionate to decreases in total gray matter. These volumetric differences were significantly related performance on standardized assessments of intelligence, including tests of both verbal and non-verbal (i.e., performance) skills. The specific pattern of association differed between the DS22q11.2 and control groups, with verbal IQ indices more strongly correlated in the DS22q11.2 group and performance IQ indices more strongly correlated in the control group. These findings are discussed, in turn, in the following section.

## Discussion

Children with DS22q11.2 exhibited a disproportionate decrease in left hippocampal volume compared to age matched controls, even after statistically controlling for group differences in overall gray matter, age, and data collection site. Children with DS22q11.2 also exhibited smaller volumes of the right hippocampus, and amygdala bilaterally, but these reductions were not disproportionate to reductions in gray matter. Consistent with previous reports, children with DS22q11.2 also scored significantly lower than age-matched controls on all indices of the cognitive assessments.

Although previous studies have suggested an association between decreased hippocampal volume and neuropsychological and behavioral characteristics [25] in children with DS22q11.2, this hypothesis has not been examined empirically. This study is the first to show associations between decreased hippocampal volume and intelligence in children with DS22q11.2. Specifically, significant correlations were observed between hippocampal volume and IQ, even after controlling for group differences in gray matter, age, and data collection site. Importantly, these differences did not differ as a function of the covariates utilized (gray matter vs. total brain volume). In contrast, amygdalar volumes were not related to cognitive measures (cf. [26]). Similar to findings of regional reduction in temporal lobe volume in children DS22q11.2 [17], significant associations were found between hippocampal volume and Verbal IQ (as indexed by VC/VCI) but not Performance IQ (as indexed by PO/PRI) in the DS22q11.2 subgroup. Previous research has suggested that hippocampal volume is strongly related to intellectual function during typical development (e.g., [30,31]). In our investigation this association held for the DS22q11.2 group in the verbal domain, which is a relative strength in this population. However, this relation did not hold in the nonverbal domain, which is known to be significantly impaired in individuals with DS22q11.2, especially in area of spatial cognition [14].

The cognitive skills subserved by the hippocampus and associated cortical regions undergo significant developmental changes during middle childhood and adolescence. Not only are the effects of genetics important to consider but hormonal and environmental effects are as well. Results in the control group presented in the current paper varied slightly from previous published investigations, which report significant associations between hippocampal volume and Verbal IQ and not between hippocampal volume and Performance IQ. We propose that this disparity may be attributable to differences in the ages and genders included in the analyses and possibly the specific IQ measurement used, as the single existing previous report included older children, was restricted to male participants only, and used a different IQ assessment [30].

Additionally, at present, we can only speculate as to why the pattern of correlations between hippocampal volume and IQ differed between children in the DS22q11.2 and control groups. It is possible that within the DS22q11.2 group the developmental trajectory of the hippocampus was altered early during neurodevelopment as either a direct result of the genetic deletion or as a downstream effect. This variation may have affected gray matter within the hippocampus (as measured in the present report) as well as wider connectivity between the hippocampus and other cortical regions. These alterations to neurodevelopment could have provided an opportunity for functional reorganization and/or compensatory mechanisms to emerge. The resulting outcome of these changes may have had a stronger effect on nonverbal cognitive domains, which are more impaired in this population compared to verbal domains, thus resulting in a significant correlation between Performance IQ (as indexed by PO/PCI) for the control group but not for the DS22q11.2 group. The complex development of connectivity between regions in the medial temporal lobe, such as the hippocampus and other cortical regions, is a source of current investigation in typically developing children [38]. It is our hope that future investigations will continue to explore not only how this connectivity is altered within DS22q11.2 population but also associate these changes with cognitive outcomes.

Finally, there are two aspects of the present investigation that limit interpretation of the results. First, multiple scanner sites were used in order to obtain data. Although scanner site was used as a covariate in all analyses to statistically control for potential effects, future investigations should limit data collection to one site. A second limitation is that, although calculation of both hippocampal and amygdalar volumes were derived from native space, whole brains were spatially transformed to a common anatomical template before gray matter, white matter, and CSF volumes were calculated. Based on previous research, it is very likely that the two groups had different global brain volumes before, as well as after, the transformation. The fact that those differences might have caused the template normalization to behave differently in the two groups cannot be ruled out. Although this issue does not directly impact volumetric calculations of the primary regions of interest (hippocampus and amygdala), the indirect effects on the covariates are unknown.

## Conclusion

Chromosome 22q11.2 deletion syndrome provides a useful model to explore specific genetic influences on brain development and resulting cognitive outcomes [17]. The findings reported here indicate that decreased hippocampal volume is associated with cognitive performance. Not only do these findings contribute to the understanding of the neuroanatomical variation in DS22q11.2, but they provide insight into the nature and possible source of the cognitive impairments associated with the syndrome. Given the known association between intellectual impairment, risk for psychosis, and reduced hippocampal volume [39,40] careful characterization of hippocampal morphology and associations with cognition *in the same participants *is essential for predicting outcomes in individuals with this syndrome and for the understanding of this neurodevelopmental disorder.

## List of Abbreviations

DS22q11.2: Chromosome 22q11.2 deletion syndrome

VC/VCI : Verbal Comprehension Factor/Verbal Comprehension Index (VCI) Wechsler Intelligence Scale for Children (WISC-III/IV)

PO/PCI: Perceptual Organization (nonverbal) Factor/Perceptual Reasoning Index (PRI) Wechsler Intelligence Scale for Children (WISC-III/IV)

FSIQ: Full Scale Intelligence Quotient Wechsler Intelligence Scale for Children (WISC-III/IV)

IQ: Intelligence Quotient

MRI: Magnetic resonance imaging

CSF: Cerebrospinal fluid

WISC-III/IV: Wechsler Intelligence Scale for Children version III/IV

## Competing interests

The author(s) declare that they have no competing interests.

## Authors' contributions

TD drafted the manuscript and supervised all management, analysis, and interpretation of the data. ZW contributed to data analysis and helped to draft the manuscript. AL provided quality assurance of volumetric MRI data and assisted with data management. TJS conceived of the study, secured funding, was responsible for its design and coordination, and helped to draft the manuscript. All authors read and approved the final manuscript.

## References

[B1] Murphy KC, Scambler PJ (2005). Velo-cardio-facial syndrome: a model for understanding microdeletion disorders.

[B2] Driscoll DA, Budarf ML, Emanuel BS (1992). A genetic etiology for DiGeorge syndrome: consistent deletions and microdeletions of 22q11. Am J Hum Genet.

[B3] Emanuel BS, McDonald-McGinn D, Saitta SC, Zackai EH (2001). The 22q11.2 deletion syndrome. Adv Pediatr.

[B4] McDonald-McGinn DM, Kirschner R, Goldmuntz E, Sullivan K, Eicher P, Gerdes M, Moss EM, Solot CB, Wang PP, Jacobs I, Handler S, Knightly C, Heher K, Wilson M, Ming JE, Grace K, Driscoll DA, Pasquariello P, Randall P, LaRossa D, Emanuel BS, Zackai EH (1999). The Philadelphia story: the 22q11.2 deletion: report on 250 patients. Genet Couns.

[B5] Niklasson L, Rasmussen P, Oskarsdottir S, Gillberg C (2001). Neuropsychiatric disorders in the 22q11 deletion syndrome. Genet Med.

[B6] Gothelf D, Presburger G, Zohar AH, Burg M, Nahmani A, Frydman M, Shohat M, Inbar D, Aviram-Goldring A, Yeshaya J, Steinberg T, Finkelstein Y, Frisch A, Weizman A, Apter A (2004). Obsessive-compulsive disorder in patients with velocardiofacial (22q11 deletion) syndrome. Am J Med Genet B Neuropsychiatr Genet.

[B7] Papolos DF, Faedda GL, Veit S, Goldberg R, Morrow B, Kucherlapati R, Shprintzen RJ (1996). Bipolar spectrum disorders in patients diagnosed with velo-cardio-facial syndrome: does a hemizygous deletion of chromosome 22q11 result in bipolar affective disorder?. Am J Psychiatry.

[B8] Bassett AS, Chow EW (1999). 22q11 deletion syndrome: a genetic subtype of schizophrenia. Biol Psychiatry.

[B9] Campbell L, Swillen A, Murphy KC, Scambler PJ (2005). The cognitive spectrum in velo-cardio-facial syndrome. Velo-Cardio-Facial Syndrome: A Model for Understanding Microdeletion Disorders.

[B10] Gerdes M, Solot CB, Wang PP, Moss EM, LaRossa D, Randall P, Goldmuntz E, Clark BJ, Driscoll DA, Jawad A, Emmanuel BS, McDonald-McGinn DM, Batshaw ML, Zackai EH (1999). Cognitive and behavior profile of preschool children with chromosome 22q11.2 deletion. Am J Med Genet.

[B11] Gerdes M, Solot C, Wang PP, McDonald-McGinn DM, Zackai EH (2001). Taking advantage of early diagnosis: preschool children with the 22q11.2 deletion. Genet Med.

[B12] Jansen PW, Duijff SN, Beemer FA, Vorstman JAS, Klaassen PWJ, Morcus MEJ, Boer JAH (2007). Behavioral problems in relation to intelligence in children with 22q11.2 deletion syndrome: a matched control study. Am J Med Genet A.

[B13] Swillen A, Vandeputte L, Cracco J, Maes B, Ghesquiere P, Devriendt K, Fryns JP (1999). Neuropsychological, learning and psychosocial profile of primary school aged children with the velo-cardio-facial syndrome (22q11 deletion): evidence for a nonverbal learning disability?. Child Neuropsychol.

[B14] Simon TJ, Bish JP, Bearden CE, Ding L, Ferrante S, Nguyen V, Gee JC, McDonald-McGinn DM, Zackai EH, Emannuel BS (2005). A multiple levels analysis of cognitive dysfunction and psychopathology associated with chromosome 22q11.2 deletion syndrome in children. Dev Psychopathol.

[B15] Bearden CE, Woodin MF, Wang PP, Moss E, McDonald-McGinn D, Zackai E, Emannuel B, Cannon TD (2001). The neurocognitive phenotype of the 22q11.2 deletion syndrome: selective deficit in visual-spatial memory. J Clin Exp Neuropsychol.

[B16] Simon TJ, Bearden CE, McDonald-McGinn DM, Zackai E (2005). Visuospatial and numerical cognitive deficits in children with chromosome 22q11.2 deletion syndrome. Cortex.

[B17] Bearden CE, Erp TGM, Monterosso JR, Simon TJ, Glahn DC, Saleh PA, Hill NM, McDonald-McGinn DM, Zackai E, Emanuel BS, Cannon TD (2004). Regional brain abnormalities in 22q11.2 deletion syndrome: association with cognitive abilities and behavioral symptoms. Neurocase.

[B18] van Amelsvoort T, Daly E, Robertson D, Suckling J, Ng V, Critchley H, Owen MJ, Henry J, Murphy KC, Murphy DGM (2001). Structural brain abnormalities associated with the deletion at chromosome 22q11. Br J Psychiatry.

[B19] Eliez S, Schmitt JE, White CD, Reiss AL (2000). Children and adolescents with Velocardiofacial Syndrome: a volumetric study. Am J Psychiatry.

[B20] Kates WR, Burnette CP, Jabs EW, Rutberg J, Murphy AM, Grados M, Geraghty M, Kaufmann WE, Pearlson GD (2001). Regional cortical white matter reductions in velocardiofacial syndrome: a volumetric MRI analysis. Biol Psychiatry.

[B21] Simon TJ, Ding L, Bish JP, McDonald-McGinn DM, Zackai EH, Gee J (2005). Volumetric, connective, and morphologic changes in the brains of children with chromosome 22q11.2 deletion syndrome: an integrative study. Neuroimage.

[B22] Eliez S, Blasey CM, Schmitt EJ, White CD, Hu D, Reiss AL (2001). Velocardiofacial syndrome: are structural changes in the temporal and mesial temporal regions related to schizophrenia. Am J Psychiatry.

[B23] Chow EW, Zipursky RB, Mikulis DJ, Bassett AS (2002). Structural brain abnormalities in patients with schizophrenia and 22q11 deletion syndrome. Biol Psychiatry.

[B24] Campbell LE, Daly E, Toal F, Stevens A, Azuma R, Catani M, Ng V, van Amelsvoort T, Chitnis X, Cutter W, Murphy DG, Murphy KC (2006). Brain and behaviour in children with 22q11.2 deletion syndrome: a volumetric and voxel-based morphometry MRI study. Brain.

[B25] Debbane M, Schaer M, Farhoumand R, Glaser B, Eliez S (2006). Hippocampal volume reduction in 22q11.2 deletion syndrome. Neuropsychologia.

[B26] Kates WRWR, Miller AMAM, Abdulsabur NN, Antshel KMKM, Conchelos JJ, Fremont WW, Roizen NN (2006). Temporal lobe anatomy and psychiatric symptoms in velocardiofacial syndrome (22q11.2 deletion syndrome). J Am Acad Child Adolesc Psychiatry.

[B27] van Amelsvoort T (2004). Brain anatomy in adults with velocardiofacial syndrome with and without schizophrenia preliminary results of a structural magnetic resonance imaging study. Arch Gen Psychiatry.

[B28] Kates WR (1997). Reliability and validity of MRI measurement of the amygdala and hippocampus in children with fragile X syndrome. Psychiatry Res.

[B29] Achenbach TM, Edelbrock C (1991). Manual for the Child Behavior Checklist and Revised Child Behavior Profile.

[B30] Schumann CMCM, Hamstra JJ, Goodlin-Jones BLBL, Kwon HH, Reiss ALAL, Amaral DGDG (2007). Hippocampal size positively correlates with verbal IQ in male children. Hippocampus.

[B31] Van Petten CC (2004). Relationship between hippocampal volume and memory ability in healthy individuals across the lifespan: review and meta-analysis. Neuropsychologia.

[B32] Nelson MD (1998). Hippocampal volume reduction in schizophrenia as assessed by magnetic resonance imaging: a meta-analytic study. Arch Gen Psychiatry.

[B33] [http://www.fil.ion.ucl.ac.uk/spm/] W Website title. http://www.fil.ion.ucl.ac.uk/spm/.

[B34] Marko Wilke VJSSKH (2002). Assessment of spatial normalization of whole-brain magnetic resonance images in children.

[B35] Ashburner J, Friston KJ (2000). Voxel-based morphometry---The methods. Neuroimage.

[B36] Robb RARA (1989). ANALYZE: A comprehensive, operator-interactive software package for multidimensional medical image display and analysis. Comput Med Imaging Graph.

[B37] Schumann CM, Hamstra J, Goodlin-Jones BL, Lotspeich LJ, Kwon H, Buonocore MH, Lammers CR, Reiss AL, Amaral DG (2004). The amygdala is enlarged in children but not adolescents with autism; the hippocampus is enlarged at all ages. J Neurosci.

[B38] Menon V, Boyett-Anderson JM, Reiss AL (2005). Maturation of medial temporal lobe response and connectivity during memory encoding. Brain Res Cogn Brain Res.

[B39] Lawrie SM, Whalley HC, Abukmeil SS, Kestelman JN, Miller P, Best JJK, Owens DGC, Johnstone EC (2002). Temporal lobe volume changes in people at high risk of schizophrenia with psychotic symptoms. Br J Psychiatry.

[B40] Wood SSJ, Pantelis CC, Proffitt TT, Phillips LLJ, Stuart GGW, Buchanan JJA, Mahony KK, Brewer WW, Smith DDJ, McGorry PPD (2003). Spatial working memory ability is a marker of risk-for-psychosis. Psychol Med.

